# Fatal vascular complications during transradial percutaneous coronary intervention: A case report

**DOI:** 10.1097/MD.0000000000021205

**Published:** 2020-07-10

**Authors:** SeongIl Choi, Joon Hee Joh, Ju Won Choe

**Affiliations:** a Department of Cardiology; b Department of Radiology; c Department of Thoracic surgery, Hanyang University Hanmaeum Changwon Hospital, Changwon-si, Korea.

**Keywords:** fatal outcome, hemorrhage, mediastinum, percutaneous coronary intervention, radial artery, therapeutic embolization

## Abstract

**Rationale:**

Vascular complications of transradial percutaneous coronary intervention (PCI) are rare and usually occur at the access site below the elbow. However, vessels along the tract of the wire or catheter can be injured at any point, causing various types of bleeding complications.

**Patient concerns:**

A 57-year-old man visited due to chest discomfort. Coronary angiography showed significant stenosis at the distal right coronary artery (RCA). Immediately after the coronary guidewire was passed through the distal RCA, he started a vigorous cough. The voice changed, dyspnea occurred within minutes, and lip cyanosis and stridor were observed. After endotracheal intubation, successful stenting of the distal RCA was achieved. He was extubated at 30 minutes after coronary stenting, but 1-hour post-extubation, his blood pressure suddenly decreased to 70/50 mmHg.

**Diagnosis:**

Mediastinal widening was newly noted on chest X-ray, and blood hemoglobin was decreased. Contrast-enhanced chest computed tomography showed mediastinal hematoma, tracheal compression, and hemothorax. Contrast extravasation was noted in the terminal branches of the inferior thyroid artery on brachiocephalic angiography.

**Interventions:**

Successful hemostasis was achieved with endovascular embolization therapy using a Tornado embolization microcoil, Gelfoam gelatin sponge, and Histoacryl glue. The next day, the mediastinal hemorrhage was drained by mediastinoscopy. The endotracheal intubation and ventilator care were maintained for 2 days, and 6 units of packed red blood cells were transfused. Antithrombotics were used to prevent stent thrombosis, and antibiotics to control infection, respectively.

**Outcomes:**

After successful hemostasis, thrombocytosis and high on-treatment platelet reactivity that disappeared at 2 weeks post-discharge were noted. Follow-up chest imaging showed the normalized mediastinal widening. At 14 months post-discharge, the patient remains healthy.

**Lessons:**

As life-threating vascular complications, such as brachiocephalic, subclavian vessel dissection, and vessel perforation in the internal mammary, costocervical, and thyrocervical arteries, can occur anytime during transradial PCI, the intervention cardiologist should be well aware of it and have the appropriate countermeasures implemented in the routine procedure.

## 1 Introduction

Transradial percutaneous coronary intervention (PCI) is a safer and more effective treatment than using the femoral artery approach because of the lower procedure-related complications. These factors together expedite patients’ return to work and daily life, thereby improving their quality of life, and have driven the universal popularization of coronary angiography (CAG). Transradial PCI has contributed to the early detection of coronary artery disease and reduced morbidity and mortality across the spectrum of cardiovascular disease. It is thus recognized as standard therapy for coronary artery disease in contemporary medicine.

The greatest advantage of transradial PCI is reduced bleeding complications. Most are minor bleedings in small vessels at the access site below the elbow and some episodes of bleeding complications associated with cardiogenic shock.^[1]^ This evidence supports a “radial-first” strategy in various current guidelines, including ST-segment elevation myocardial infarction (STEMI).^[2]^ Despite an extremely low incidence of complications, often trivialized in the literature,^[3]^ the transradial approach is not free of life-threating vascular complications, particularly brachiocephalic artery perforation,^[3,4]^ subclavian vessel dissection,^[3,5–9]^ and vessel perforation in the internal mammary artery,^[1]^ costocervical trunk,^[10]^ and thyrocervical trunk.^[11,12]^ Thus, the intervention cardiologist must be well-informed about the life-threating complications specific to the radial approach and have countermeasures implemented in advance. We reported a case of inferior thyroid arterial bleeding that resulted in tracheal compression and respiratory failure during transradial PCI. The patient was successfully treated using endovascular embolization and mediastinotomy drainage.

## 2 Case report

**Figure 1:**
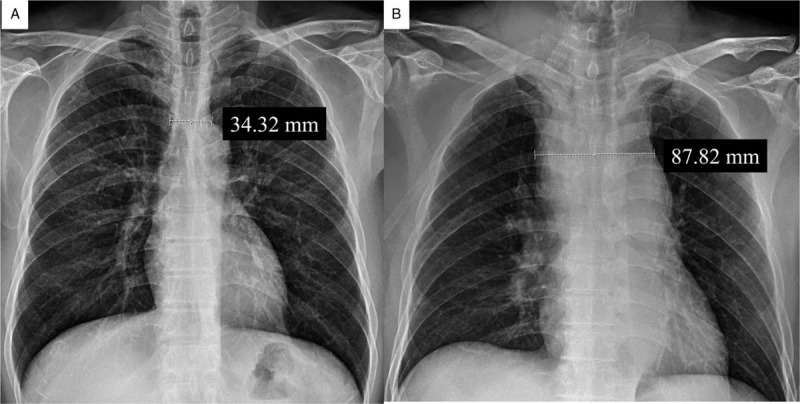
Chest X-ray. **A.** The pre-interventional image shows the normal range of mediastinum and chest. **B.** The post-interventional image shows the newly developed mediastinal widening. The numbers indicate the mediastinal size just above the aortic arch level.

**Figure 2:**
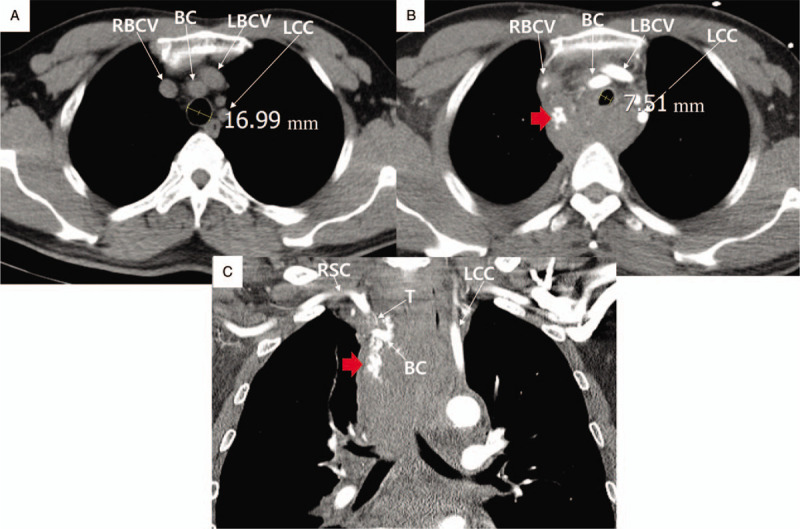
Chest computed tomography. **A.** The pre-interventional image (1 year ago) shows the normal range of mediastinum, trachea, and lung. **B.** The post-interventional image after coronary stenting shows mediastinal hematoma, tracheal compression, and hemothorax, suggesting brachiocephalic arterial rupture. **C.** The post-interventional coronal section image after coronary stenting shows the extravasated blood around the brachiocephalic trunk region and a large mediastinal hematoma. A red arrow indicates the active bleeding site. The numbers indicate the tracheal diameters. BC = brachiocephalic artery, LBCV = left brachiocephalic vein, LCC = left common carotid artery, RBCV = right brachiocephalic vein, T = right inferior thyroid artery.

**Figure 3:**
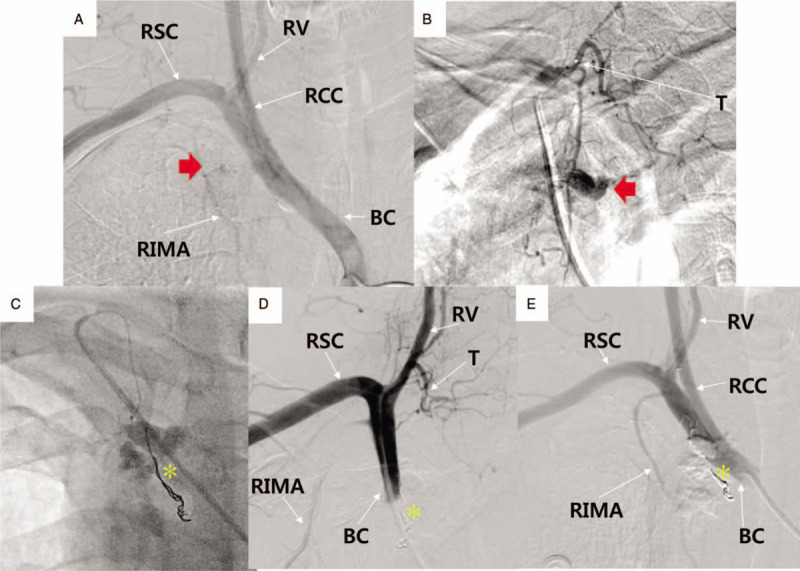
Peripheral angiography. **A.** The pre-interventional image using dye injector distinguishes the contrast-staining sites from the normal vascular structure, suggesting the extravasated blood by the vessel perforation. **B.** The pre-interventional image using a microcatheter shows the bleeding from the terminal portion of the right inferior thyroid artery and vessel anastomosis. **C.** Successful hemostasis was achieved by endovascular embolization using a Tornado microcoil, Gelfoam gelatin sponge, and Histoacryl glue. **D.** The post-interventional image after embolization shows the fully-occluded blood flow below the proximal portion of the inferior thyroid artery by embolization therapy. **E.** The post-interventional image after embolization shows the absence of contrast-staining sites. Instead, Tornado microcoils and Histoacryl glue are newly noted. A red arrow indicates the active bleeding site. An asterisk indicates the Tornado microcoils and glue remnants applied to the bleeding vessels. BC = brachiocephalic artery, RCC = right common carotid artery, RIMA = right internal mammary artery, RSC = right subclavian artery, RV = right vertebral artery, T = right inferior thyroid artery.

A 57-year-old man visited due to chest discomfort and right shoulder pain, 2 weeks ago. A health checkup a week ago revealed no abnormal finding. He had a history of anterior wall STEMI 18 months ago. He underwent coronary stenting at the proximal left anterior descending artery and received intra-aortic balloon pump therapy for cardiogenic shock. Antiplatelet agent (aspirin 100 mg daily, ticagrelor 90 mg twice daily) and statin (rosuvastatin 20 mg daily) were taken, and smoking was currently maintained. For the coronary anatomy evaluation, CAG was performed under fluoroscopic guidance, using the right radial artery, a 4 French (F) sheath, a 0.035″ × 180 cm, angled, J-type Radifocus hydrophilic guidewire (Terumo, Tokyo, Japan), and a 4 F Tiger II Outlook 100-cm coronary catheter (Terumo). CAG showed no significant in-stent restenosis at the proximal left anterior descending artery but significant stenosis at the distal right coronary artery (RCA). For PCI of the distal RCA, a 4 F sheath was replaced with a 6 F sheath, and RCA was engaged under fluoroscopic guidance, using a 0.014″ × 195 mm ATW steerable coronary guidewire (Cordis, MI) and a 6 F Vista Brite Tip Judkins right 4.0 coronary catheter (Cordis). Immediately after the ATW coronary guidewire was passed through the distal RCA, the patient began coughing vigorously in several consecutive episodes and exhibited facial redness. PCI was reserved until he was stabilized. Unexpectedly, however, the voice changed, dyspnea occurred within a few minutes, and lip cyanosis and stridor were observed. Initially, a dye-induced anaphylactic reaction was suspected. Corticosteroid and antihistamine (Pheniramine) were injected intravenously, and endotracheal intubation was successfully performed by the anesthesiologist. After endotracheal intubation, 100% oxygen saturation was recovered, vital signs stabilized, and alert mentation was observed. Balloon angioplasty and coronary stenting were performed using a 2.5 mm × 15 mm balloon catheter (Lacrosse NSE, Goodman Medical, Ireland) and 3.25 mm × 18 mm Xience Sierra stent (Abbott, Santa Clara, CA), respectively. PCI was terminated successfully after high-pressure balloon angioplasty using a 3.25 mm × 8 mm non-compliant balloon (Powered Lacrosse 2, Goodman Medical). The intubated patient was moved to the intensive care unit, for ventilator support and close hemodynamic monitoring. Because spontaneous respiration, obey command and 100% oxygen saturation were maintained, he was extubated 30 minutes after PCI. At 1-hour post-extubation, the oxygen saturation was maintained above 97%. However, his blood pressure suddenly decreased to 70/50 mmHg and recovered to 100/70 mmHg by intravenous fluid hydration and dopamine infusion. Despite the normal ranges of blood pressure and oxygen saturation, he was agitated and complained of chest discomfort. Transthoracic echocardiography, electrocardiography, chest X-ray, and blood test were performed. On the transthoracic echocardiography and electrocardiography, there was no significant interval change from previous findings, but a markedly widened superior mediastinum was newly noted on chest X-ray, and blood hemoglobin was reduced from 15.2 to 13.0 g/dL, suggesting a mediastinal hemorrhage (Fig. 1A and B). As the contrast-enhanced chest computed tomography (CT) scan performed immediately also showed contrast extravasation surrounding the brachiocephalic artery, and a mediastinal hematoma compressing the trachea and hemothorax, hemorrhagic shock by brachiocephalic vessel rupture was strongly suspected (Fig. 2A–C). First, for the perforating vessel closure, brachiocephalic angiography was performed using the right femoral artery. The innominate, subclavian, and vertebral arteries were intact, whereas contrast extravasation was noted in the terminal portion of the inferior thyroidal artery of the thyrocervical trunk (Fig. 3A and B). Endovascular embolization therapy was performed successfully using a 3 × 2 mm Tornado embolization microcoil (Cook Medical, Bloomington, IN), Gelfoam gelatin sponge (Medical Impact, Bucheon, Korea), and Histoacryl glue (B/Braun, Barcelona, Spain), which extinguished the extravasation blood (Fig. 3C–E).

The next day (hospitalization day 2), the mediastinal hemorrhage was drained by mediastinoscopy, to relieve the trachea compression and fibrosis due to extravasated blood. In total, 1.5 L of blood was drained from the mediastinum and hemothorax. The endotracheal intubation and ventilator care were maintained for 2 days, due to severe tracheal compression by the mediastinum hematoma. After 6 units of packed red blood cells were transfused, hemoglobin was maintained at 9.6 g/dL, and vital signs and oxygen saturation were stabilized. The antithrombotic agent ticagrelor was maintained at 60 mg twice daily, to prevent stent thrombosis. Additionally, antibiotics were used for infection control.

**Figure 4:**
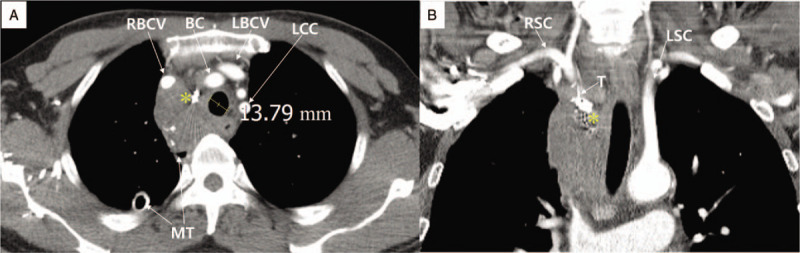
Chest computed tomography. **A.** The 1-week image after coronary stenting shows the decreased amount of mediastinal hematoma and the increased tracheal diameter. **B.** The 1-week coronal section image after coronary stenting shows the occluded terminal branch of the right inferior thyroid artery, with endovascular embolization using a microcoil and glue but the mediastinal hematoma remained. An asterisk indicates the Tornado microcoils and glue remnants applied to the bleeding vessels. BC = brachiocephalic artery, LBCV = left brachiocephalic vein, LCC = left common carotid artery, LSC = left subclavian artery, MT = mediastinal drainage tube, RBCV = right brachiocephalic vein, RCS = right subclavian artery, T = right inferior thyroid artery.

On day 4, airway patency was improved, and extubation was performed. As hemoglobin was maintained at 9.7 g/dL, aspirin (100 mg daily) plus ticagrelor (60 mg twice daily) was administered. The follow-up chest CT on day 7 showed the mediastinum hematoma had decreased in size (Fig. 4A and B). On day 9, the mediastinal drainage had decreased to below 50 mL, and the drainage tube was removed. Meanwhile, antiplatelet therapy, as measured by the Multiplate Analyzer (Roche Diagnostics, Mannheim, Germany), showed a high on-treatment platelet reactivity (56; normal range; 1–38) and also thrombocytosis (platelet count; 799,000/μL), so the daily ticagrelor dose increased from 60 mg twice to 90 mg twice. On day 10, the patient was discharged with medication, including antiplatelet agents and rosuvastatin.

**Figure 5:**
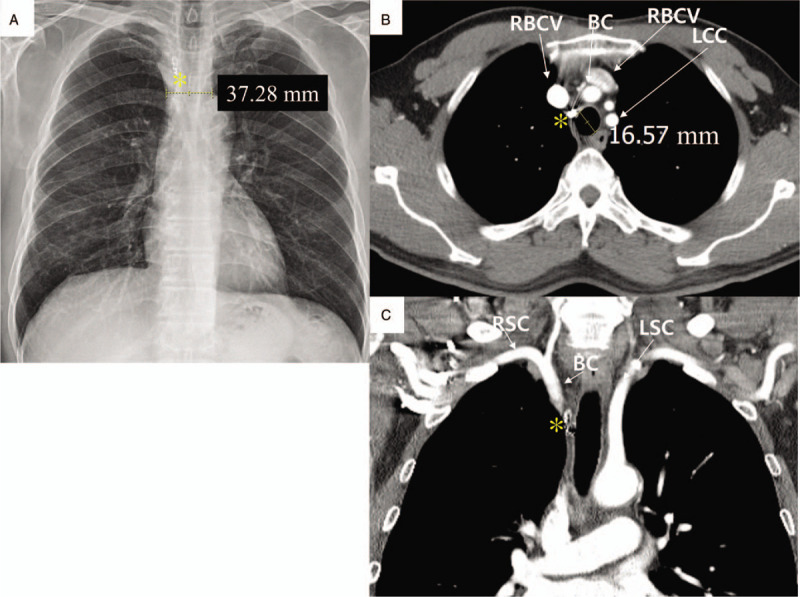
Post-discharge follow-up study. **A.** Chest X-ray image at 1-month post-discharge shows the normalization of mediastinal widening. The number indicates the mediastinal size just above the aortic arch level. **B.** Chest computed tomography image at 2 months post-discharge shows the recovery to the normal range of mediastinal and trachea, except for remnants of embolization therapy. The numbers indicate the tracheal diameters. **C.** Chest computed tomography coronal section image at 2 months post-discharge shows the occluded terminal branch of the right inferior thyroid artery, with endovascular embolization and complete resolution of the mediastinal hematoma. An asterisk indicates the Tornado microcoils and glue remnants applied to the bleeding vessels. BC = brachiocephalic artery, LBCV = left brachiocephalic vein, LCC = left common carotid artery, LSC = left subclavian artery, RBCV = right brachiocephalic vein, RCS = right subclavian artery.

Two weeks post-discharge, high on-treatment platelet reactivity had disappeared, and the follow-up chest imaging showed the normalized mediastinal structure, except for embolization remnants (Fig. 5A–C). At 14 months post-discharge, the patient remains healthy.

## 3 Discussion

Our case of inferior thyroid arterial bleeding, which resulted in a mediastinal hemorrhage complicating tracheal compression and respiratory failure during transradial PCI, was successfully treated with endovascular embolization and mediastinotomy drainage. To our knowledge, this is the third case report of inferior thyroidal arterial bleeding (including thyrocervical truck) during transradial PCI,^[11,12]^ the twelfth report of mediastinal hemorrhage or hematoma,^[3–6,8–14]^ and the fifth description of tracheal compression requiring endotracheal intubation^[4,10–12]^ Moreover, it is the first case report of inferior thyroid bleeding during transradial PCI using an angled J-type hydrophilic wire, and succeeding in bleeding control using a microcoil, gel sponge, and glue.

The most notable feature of this case was the extravascular bleeding in the small branch vessel that was not closely associated with CAG or PCI during the routine procedure. Previously, the use of a hydrophilic wire, rough catheter manipulation, and extravasated bleeding during transradial PCI in patients with severe vessel tortuosity have been reported, but no case was ever reported in a routine process performed without difficulty as scheduled.^[3,4,8]^ In our case, several severe coughs occurred just before the event, which was also observed in Smilowitz's case.^[4]^ However, it is unclear whether extravascular bleeding or mediastinal hemorrhage was ascribed to those severe coughs alone. Conversely, we assumed that the cough was more likely a symptom of mediastinal hemorrhage.

Both rapid diagnosis and prompt treatment are the most important factors to determine the survival or prognosis of mediastinal hemorrhage and tracheal compression by vessel perforation during the transradial approach. Although an important clue is the mediastinal widening on chest X-ray, an emergency contrast-enhanced chest CT scan should be performed to identify the location of the lesion, assess the extent of bleeding, and establish the therapeutic strategy for hemostasis. Once the bleeding focus is confirmed on chest CT, the peripheral angiography should be performed immediately. Although a few cases initially achieved successful treatment with conservative therapy alone,^[3,8,10,12,13]^ most cases reported progressive bleeding and deteriorated patient status, which led to the emergency peripheral intervention. Therefore, both prompt peripheral angiography and endovascular intervention are recommended.

Meanwhile, because the manual dye injection may not be definitive to confirm the bleeding,^[5]^ especially given the small amount of bleeding by branch vessel perforation, the first peripheral angiography image should be confirmed by cine angiography using a dye injector, instead of manual dye injection. Thus, we initially placed the catheter in the brachiocephalic and subclavian arteries, confirmed the contrast extravasation from the inferior thyroid artery of the thyrocervical trunk through cine angiography using a dye injector and then selected the perforating vessel (right thyrocervical trunk). Finally, the bleeding focus was identified by manual dye injection using the catheter and Asahi Masters Parkway Soft microcatheter (Asahi, Japan). In principle, a covered graft stent (for a large artery), coil embolization (for a medium artery), and gel sponge and glue can be used (for a small artery). As our case had bleeding from the small branch vessel, successful hemostasis was achieved using a microcoil, gel sponge, and glue.

Another point is that the mediastinal hemorrhage caused by vessel perforation shows symptoms of tracheal obstruction and signs of respiratory failure, which is difficult to differentiate from a dye-induced allergic reaction or a bronchial asthma attack. It is particularly difficult to discriminate those causes in patients with endotracheal intubation due to tracheal obstruction and respiratory failure. We initially diagnosed as the dye-induced allergic reaction and prescribed intravenous steroid and antihistamine injection. Those same treatments were undertaken in Smilowitz's case.^[4]^

A third noteworthy point is the importance of a multi-department team approach. The operator cannot manage alone because the mediastinal hemorrhage by vessel perforation complicates tracheal compression rapidly and, also, progresses to respiratory failure. In other words, because airway patency should be maintained, the perforating vessel should be identified and closed, and additional treatments for mediastinal hematoma should also be needed, systematic work-sharing and close collaboration are inevitable in individual expert fields. In our case, the anesthesiologist maintained the airway patency, the interventional cardiologist performed PCI, the intervention radiologist closed the perforated vessel, and the thoracic surgeon managed the mediastinal hematoma.

## 4 Conclusion

Vascular complications of the transradial approach are rare and usually occur at the access site below the elbow. However, vessels along the tract of the wire or catheter can also be injured at any point, causing various types of bleeding complications. In particular, as life-threating vascular complications, such as brachiocephalic, subclavian vessel dissection, and vessel perforation in the internal mammary, costocervical, and thyrocervical arteries can occur anytime during transradial PCI, the intervention cardiologist should be well aware of it and have countermeasures prepared in routine transradial PCI. We reported a case of inferior thyroid arterial bleeding that caused tracheal compression and respiratory failure during transradial PCI. It was successfully treated by endovascular embolization and mediastinotomy drainage.

## Author contributions

**Conceptualization:** SeongIl Choi.

**Data curation:** Joon Hee Joh, Ju Won Choe.

**Investigation:** SeongIl Choi.

**Methodology:** SeongIl Choi.

**Visualization:** Joon Hee Joh.

**Writing – original draft:** SeongIl Choi.

**Writing – review & editing:** Joon Hee Joh, Ju Won Choe.
